# Heterogeneity Effects in Highly Cross-Linked Polymer Networks

**DOI:** 10.3390/polym13050757

**Published:** 2021-02-28

**Authors:** Gérald Munoz, Alain Dequidt, Nicolas Martzel, Ronald Blaak, Florent Goujon, Julien Devémy, Sébastien Garruchet, Benoit Latour, Etienne Munch, Patrice Malfreyt

**Affiliations:** 1 Manufacture Française des Pneumatiques Michelin, Site de Ladoux, 23 Place des Carmes Déchaux, France CEDEX 9, 63040 Clermont-Ferrand, France; gerald.munoz@michelin.com (G.M.); sebastien.garruchet@michelin.com (S.G.); benoit.latour@michelin.com (B.L.); etienne.munch1@michelin.com (E.M.); 2 Institut de Chimie de Clermont-Ferrand, CNRS, SIGMA Clermont, Université Clermont Auvergne, 63000 Clermont-Ferrand, France; ronald.blaak@uca.fr (R.B.); florent.goujon@uca.fr (F.G.); julien.devemy@uca.fr (J.D.); patrice.malfreyt@uca.fr (P.M.)

**Keywords:** elastic network model, heterogeneity, polymers

## Abstract

Despite their level of refinement, micro-mechanical, stretch-based and invariant-based models, still fail to capture and describe all aspects of the mechanical properties of polymer networks for which they were developed. This is for an important part caused by the way the microscopic inhomogeneities are treated. The Elastic Network Model (ENM) approach of reintroducing the spatial resolution by considering the network at the level of its topological constraints, is able to predict the macroscopic properties of polymer networks up to the point of failure. We here demonstrate the ability of ENM to highlight the effects of topology and structure on the mechanical properties of polymer networks for which the heterogeneity is characterised by spatial and topological order parameters. We quantify the macro- and microscopic effects on forces and stress caused by introducing and increasing the heterogeneity of the network. We find that significant differences in the mechanical responses arise between networks with a similar topology but different spatial structure at the time of the reticulation, whereas the dispersion of the cross-link valency has a negligible impact.

## 1. Introduction

Polymer melts and gels are disordered systems with highly heterogeneous properties at the microscopic scale. These heterogeneities explain for example the large dispersion of mechanical stresses at the point of breaking observed in batches of identical macroscopic samples [1,2]. Understanding in more details the origin of the dispersion of the local properties can therefore help to improve the reliability and the performance of polymer materials (stress vs. strain, fracturing, …). Some of the heterogeneities are temporary and caused by random thermal motions, whereas others have a structural cause. In general, they impact not only the average macroscopic properties, but also the dispersion therein.

The main goal of this article is to compare the impact of different sources of heterogeneity on the mechanical response at the microscopic and macroscopic scales. By using a simple and generic model, we present a way to progressively introduce heterogeneity in the structure and topology, and we study the corresponding impact on the mechanical response. We hope that such a study can guide the design of new experimental networks and can highlight the importance of the network generation process in simulations.

Many experimental studies have investigated parameters that affect the network structure: the cross-link density [3], cross-link valency [4], chain length [5] and topology [6,7]. In most studies only the average value of the structural parameter is varied [8], but the impact of their dispersion is important as well [9]. A few experimental studies are specifically dedicated to the impact of defects or imperfections in regular polymer lattices [10,11]. Some others focus on the detection of the heterogeneity of the network in the samples [12,13].

Recent works have also investigated the impact of heterogeneity from a theoretical point of view. These works mostly deal with topological heterogeneity due to the formation of loops in random networks [14,15,16,17]. These studies aim at understanding the impact of topology on the elastic mechanical response or on the gel point.

To some extent, the mechanical properties of polymer materials can be predicted by using simulations. At the millimeter scale, the material can be described by continuum approach and could be simulated using finite element methods, provided reliable constitutive equations are known [18,19,20,21]. These constitutive equations are formulated in terms of averaged parameters. Accounting for the impact of structural heterogeneities is often implemented at the expense of an increase in the number of parameters. In order to be able to extrapolate results and properties to outside the described regime, it is desirable that the constitutive equations are obtained via a bottom-up approach from models at the microscopic scale. Often, these models focus only on average properties and like-wise assume that average input parameters are representative for the ensemble [22]. However, an approach with models based on averaged input parameters easily lacks the ability to describe essential properties emerging from correlations between different local properties [23].

In order to take the effect of local heterogeneities into account, the models at the smaller scale have to sample one or more distributions of input properties. This can be done at the atomic or coarse-grained scale using molecular dynamics simulations [24,25,26]. However, such simulations are limited to short times and small system sizes and are therefore not representative of the whole range of input parameters [27]. Moreover, in these simulations, as well as in experiments, the respective contributions of the heterogeneities cannot be separated.

Fuse network models propose a way to treat the impact of local heterogeneities [28,29]. The corresponding simulations have the merit of their simplicity, like other studies of interest [30], but cannot be regarded as realistic models of a polymer network.

In this article, we use an intermediate-scale model for an elastic polymer network that is similar to EPNET [31]. We illustrate that this type of model allows us to simulate mechanically realistic (experimentally informed or parameterized bottom-up) elastomers and gels, while leaving enough flexibility for a parameterization of the sources of heterogeneities.

In Section 2, we introduce the different sources of heterogeneity that can be varied, together with their experimental counterparts. This is followed by describing the numerical model that we have employed and in Section 3 we present the various results that have been obtained. We finish with a discussion of the main results and conclusions.

## 2. Materials and Methods

The system consists of a network of phantom chains inside a cubic simulation box. The custom parameters of the system are the box size *a*, the number of reticulation nodes, the total number of monomers (or Kuhn segments), their size (Kuhn length *b*) and the total number of polymer chains. These parameters could be tuned to represent a specific polymer with a given cross-link density and connectivity, although this is not required for the purpose of this paper. The level of heterogeneity of the system can be adjusted as we will see later.

After the system is prepared and equilibrated at a given temperature *T*, the box is slowly stretched in a quasi-static manner in the *z* direction and shrunk in the *x* and *y* directions so as to keep a constant volume. The deformation ratio is defined by $\lambda =\frac{a(t)}{a(0)}$.

At each step, the force F on each chain can be extracted and used to compute a local and a global stress σ. We are mostly interested in the force distribution resulting from the system heterogeneity, and in the excess normal stress in the stretch direction $\sigma_{z}$. The macroscopic mechanical behavior is characterizes by the modulus, which is for us the ratio $\frac{\sigma_{z}}{\lambda }$, and by the finite extension $\lambda_{\max}$, which is the maximum deformation ratio of the network, at which $\sigma_{z}$ diverges.

### 2.1. Sources of Heterogeneity

The heterogeneity that is experimentally found in polymer networks can have several different origins. This heterogeneity can stem from:

- Randomness of the chain composition in copolymers. Here we restrict ourselves to homopolymers, so that all chains have the same chemical composition between reticulation nodes.
- Polydispersity of the polymer chains between reticulation nodes, which we call “segments”. Experimentally, it is possible to control the segment polydispersity, for example by using telechelic polymer chains, resulting in a monodisperse sample. An alternative approach is to reticulate long polymer chains at random positions, which results in a geometric distribution of segment lengths [32,33,34]. In the simulations, the segment length distribution can be varied at will. This source of heterogeneity will be studied by using normal distributions of segment lengths with various standard deviations.
- Connectivity distribution. This is the number of arms attached to reticulation nodes. In experiments the connectivity can be controlled by using chemical cross-linkers of different functionalities. In general, the functionality is uniform in the sample unless a mixture of cross-linkers is used. In the case of physical cross-links, as in some gels or thermoplastic elastomers, the functionality of the reticulation nodes may be non-uniform. In our simulations, it is straightforward to choose the average connectivity of the reticulation nodes and to mix nodes of different connectivity.
- Topology of the network. The graph structure associated to the reticulation nodes can be random or regular as in a crystalline lattice. In regular structures every node is equivalent, whereas in random structures some nodes may be more “central” than others, even though the connectivity is uniform. Experimentally, the structure is often random, but it is also possible to form crystalline arrangements of reticulation nodes using block copolymers [35,36] or regular crosslinkers [10]. In this paper, we will consider both regular and random network topologies.
- Uniformity of the density of reticulation nodes describes the spatial proximity of the nodes. This source of heterogeneity is related to the network topology in a non-trivial way. It is possible to partially control the spatial distribution of the cross-links in experiments by adjusting the dispersion of the catalyst during the reticulation reaction [37]. In our simulations the spatial distribution of the nodes can be controlled in an indirect fashion. If we initially distribute the nodes with a given uniformity, they will move during the equilibration stage to new positions relaxing the mechanical constraints. They will, however, typically remain close to their original position.

In the following, the various sources of heterogeneity will be examined and, when feasible, their respective contributions will be treated individually. Note that is not always possible, for instance one cannot modify the network connectivity without changing its topology or to change the topology without affecting the spatial distribution of the reticulation nodes.

**Figure 1:**
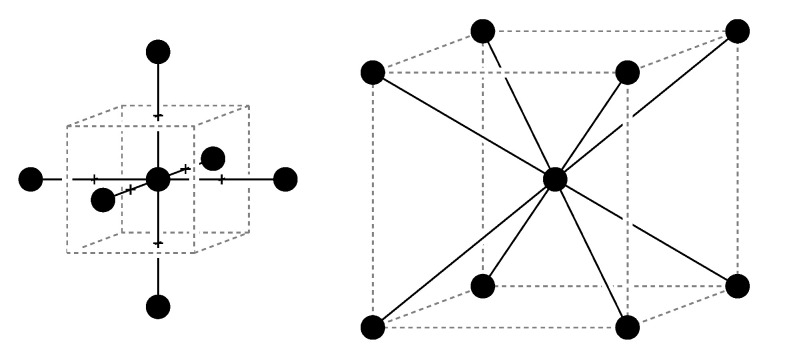
Unit cell of the six chain model (**left**) and eight chain model (**right**). The nodes represent reticulation points and the bold lines correspond to the polymer segments. These systems are labelled A.6 and A.8 in the following and correspond, respectively, to a simple cubic and to a body centered cubic lattice. All nodes are equivalent and connected to all their nearest neighbors.

Beforehand, we introduce two reference systems with uniform segment lengths, and regular network topology and spatial structure of nodes. For this purpose, we use the six chain and eight chain models which are standard models of polymer networks [18,20,38]. These models are simple enough so that we can investigate the effect of different sources of heterogeneity. Having two reference models allows to compare different connectivity, structure and topology from the beginning. These two models are known to behave differently under uniaxial and biaxial extension [22]. They are a good illustration of the constitutive equations used in finite elements methods, due to their robustness and the calculation cost advantages they provide. Nonetheless, they suffer from a non-uniform distribution of segment orientations that leads to a failure in modeling complex modal solicitations. Their structures are schematically represented in Figure 1. Both the six and the eight chain models are characterized by a number of monomers per chain *n* and a cross link density ν. Their regular topology makes that each of their nodes is equivalent. In these reference systems, the orientation of the segments is not uniformly distributed, so that the mechanical response depends on the direction of stretching. In addition, depending on their orientation, segments will experience different tension under strain, even though the system is perfectly regular.

### 2.2. Description of the Numerical Model

We have developed a coarse-grained model of polymer networks at the scale of topological constraints. This network consists of reticulation nodes (cross-links) that are connected by virtual polymer segments in an orthorhombic box with periodic boundary conditions. The nodes interact with their connected neighbors through entropic forces along the end-to-end vectors of the polymer segments. The model does not distinguish whether two segments belong to the same or to different polymer chains. Therefore in the following we will use the terms chain and segment interchangeably. Chains are characterized by the number of Kuhn segments from which they are build. These numbers are drawn from a target distribution under the constraint of the imposed density of the simulated system. It is also assumed that there are no pending chains and that each node has a minimum of 3 connected neighbors.

The system is characterized by the Kuhn length *b* of the polymer and the temperature *T* of the simulation. The nodes are subjected to random Langevin forces, which model the thermal motion of the cross-links. The simulations are performed with a home-made code, with a number of chains between 5184 and 16,384. We verified and confirmed that these numbers of chains are large enough, so that the estimated errors in curves and in the other results we present are negligible for moderate strain and do not affect the conclusions.

The network generation procedure is inspired by Hanson’s work [39] and is only based on the definition of conditional probabilities [40,41]:

(1) P(n,r)=P(n)P(r|n),

where P(n) is the probability that a network chain contains *n* monomers (polydispersity), and $P\left(r|n\right)$ the conditional probability distribution function for a chain of length *n* monomers to have an end-to-end vector r.

We start by placing the reticulation nodes in the simulation box either randomly or at prescribed positions. The number of nodes is chosen to match the experimental densities of cross-links ( $2\times 10^{19}cm^{-3}$ up to $10\times 10^{19}cm^{-3}$ depending on the polymer). A connectivity, which is the number of connection slots, is assigned to each node. Slots are initially connected randomly to any other free slot by a virtual polymer segment containing the average number of (Kuhn) monomers irrespective of (1). Note that the number of monomers in the simulation box is fixed by the target density of the total system and that the mean of P(n) should coincide with the average number of monomers per chain. Then, in order to satisfy (1), a pair of chains (i,j) is selected at random. For every possible reconnection of slots and exchange of monomers between the pair of chains, the probability *P* is computed by assuming independent chains using

(2) $$P\propto P\left(n_{i},r_{i}\right)\;P\left(n_{j},r_{j}\right).$$

One of these combinations is chosen accordingly and the process is iterated by continuing the same procedure until a stationary state is obtained. Note that the connectivity of the nodes, the number of chains and the total number of monomers are unaffected by these exchanges, and hence that also the average number of monomer per chain remains constant. This algorithm can easily be parallelized for efficiency and we refer to this process as the equilibration of the topology. Alternatively, the nodes can be connected to their nearest neighbors when the initial positions form a regular lattice. The algorithm flow chart is available in the Figure S1.

The chains are supposed to conform to the Gaussian chain model, which is valid in the large *n* limit [40]. The probability distribution function (pdf) that a chain of *n* Kuhn segments has an end-to-end distance r is given by:

(3) $$P\left(r|n\right)={\left(\frac{3}{2\pi \;b^{2}\;n}\right)}^{3/2}\exp\left(-\frac{3\;r^{2}}{2\;b^{2}\;n}\right),$$

where *b* is the Kuhn length of the particular elastomer under consideration. The probability is pruned to 0 for over-stretched chains (r>nb). For P(n), which represents the polydispersity of the chains, we restricted ourselves in this study to either uniform chain lengths or normal distributions.

The dynamics of the network, that is the motion of the nodes, is simulated by using Brownian dynamics, where the total force on each nodes is the sum of the chain forces (acting along the end-to-end vector) and a noise term (random thermal displacement).

(4) $$\gamma \;V_{i}=\sum_{j,\;\mathrm{neighbors}\;\mathrm{of}\;i}F_{j\to i}+\sqrt{2\;\gamma \;k_{B}T}\;R_{i}$$

where γ is the friction coefficient, R a random Gaussian variate with zero-mean and unit variance. $k_{B}$ is the Boltzmann constant and *T* is the absolute temperature. Since we run the simulations in the quasistatic limit, the precise value of the friction coefficient has no impact, as we have checked. The attractive force between connected nodes is assumed to be fully entropic with a finite extension and which can be described by an inverse Langevin form ($L^{-1}$). In practice, we have implemented the approximation of Cohen [42] for simplicity and which is a good approximation of $L^{-1}$. Within this approach, the elastic force can be expressed as:

(5) $$F_{j\to i}=-x\;\frac{k_{B}T}{b}\;\frac{3-x^{2}}{1-x^{2}}\;r_{j\to i}$$

where *x* is the ratio $\frac{r}{r_{max}}$, with *r* the end-to-end distance, and $r_{max}=n\;b$ the extended chain length. These forces are also used to compute the stress tensor in the simulation box using the Irving-Kirkwood formula [43]. We note that this inverse Langevin approximation is not the most accurate form (maximum relative error equal to 4.9%). This level of approximation is sufficient for the paper goal. Better approximations exist and could have been used instead [44].

In order to study the system under deformation, the dimensions of simulation box can be modified by changing the length of the 3 axes in small constant increments, while keeping the total volume constant. It was confirmed that the deformations are performed in the quasi-static limit, by checking that no difference is observed for half the deformation rate. In addition, without allowing chains to break, the maximum strain is determined by the shortest percolating path in the strain direction

(6) $$\lambda_{max}=\frac{N_{seg}\;n\;b}{a}$$

where $\lambda_{max}$ is the maximum macroscopic deformation ratio, $N_{seg}$ is the number of segments constituting the shortest percolating path and *a* the initial dimension of the simulation box along the stretch axis. With this definition, we can predict the $\lambda_{max}$ value for a system knowing the “length” of the shortest path (Figure S2). In our simulations, the shortest percolating path consists typically of about 12 chains and hence the maximum strain can only take a few discrete values, which is a finite size effect. In the following we mostly focus on small deformations (λ≲3), where the discreteness of $\lambda_{max}$ has negligible impact.

## 3. Results

To quantify the heterogeneity at different levels, we can make use of several indicators: node centrality, local nodes density and force distribution. In graph theory, the node centrality quantifies the importance of the nodes in the network (from a topological point of view). There are many ways to define node centrality, some of which have been used to study networks of force chains in granular packing [45]. We have chosen to use the “subgraph centrality”, because it is general enough and applies also to particular networks like double networks (non connected graph). This definition of centrality is based on the number of closed walks of all lengths that start and end at the node of interest [46]. For the implementation we have employed the Python library NetworkX to compute the sub-graph node centrality [47].

In order to study the local node density and quantify how evenly nodes are distributed in space, we use the number of neighbors, which is defined as all nodes within a cutoff distance. In practice, a kernel density estimation based on the position of nodes was used with a characteristic length equal to $\rho^{-1/3}$, ρ being the number density of reticulation nodes in the simulation box. The local density values in this study are calculated by means of KernelDensity-function from the Python package Scikit-learn [48].

The forces in every chain are stored during Brownian dynamics simulation for post-treatment. We analyze the distributions of the logarithm of squared forces, which is a more convenient choice than the magnitude of the forces themselves. The distribution of forces are computed at rest and at low strain values, to determine the effect of the sources of heterogeneity before the finite extension takes over as the main contributor to the macroscopic mechanical behavior.

**Figure 2:**
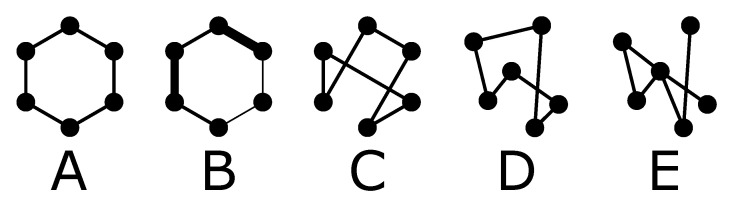
Schematic representation of the system labels. (**A**): regular lattice, (**B**): regular lattice with polydispersity, (**C**): regular structure with random topology, (**D**): random structure and topology, (**E**): random structure and non-uniform connectivity.

We label the systems using a letter followed by a number representing the average connectivity. The key differences between different systems A to E are schematically represented and summarised in Figure 2, by means of a simple 6-node graph of spatially regular/randomly distributed nodes, regular/irregular topology, and fixed/poly-disperse chain length (different line thickness) or connectivity. For the connectivity 6 and 8, the letter A refers to the reference system (six chain or eight chain models). Systems of type B are similar to A, but include polydispersity in the chain lengths. The index C corresponds to systems like A without polydispersity, but that have a random topology. This means that the initial positions of the nodes are on the same regular lattice as in the reference systems, but the chains are allowed to connect according to (1) to other nodes than just their first neighbors. Systems of type D have, like those of type C, a random topology, but now the initial position of the nodes are distributed uniformly inside the simulation box, rather than on a regular lattice. The last classification E, adds a non-uniform connectivity to systems of type D.

We note that numerically, it is possible to change progressively from A to C by truncating the equilibration of the topology earlier, but the probability distribution of the chain lengths would not yet follow (1). It is also possible to change progressively from C to D, by increasing the magnitude of the random displacements given to the initial positions of the nodes. There are two additional systems we consider. A different reference system A.4 consists of two interleaved regular diamond lattices. The other is the system C.4 that is similar to A.4, except that chains are connected randomly to any of their eight nearest neighbors. For all systems, except the ones with regular lattices topology (A.4, A.6, A.8, B.6, B.8 and C.4), the distribution of chain orientations after mechanical equilibration is uniform. A summary of the different systems and their main characterisations is provided in Table 1.

### 3.1. Polydispersity

First, we investigate the effect of polydispersity described by a normal distribution of chain lengths (Figure S3), while keeping the regular structures of the six and eight chains as reference. The standard deviation of the chain length is increased from 0 in the reference models A.6 and A.8 to 5 in the polydisperse systems B.6 and B.8. As expected, the centrality of all nodes remains the same, because the topology of the networks is not modified, and also the local node density does not change on increasing the polydispersity. This means that the presence of shorter and longer chains does not affect significantly the spatial correlations of positions of cross-links at short range.

**Figure 3:**
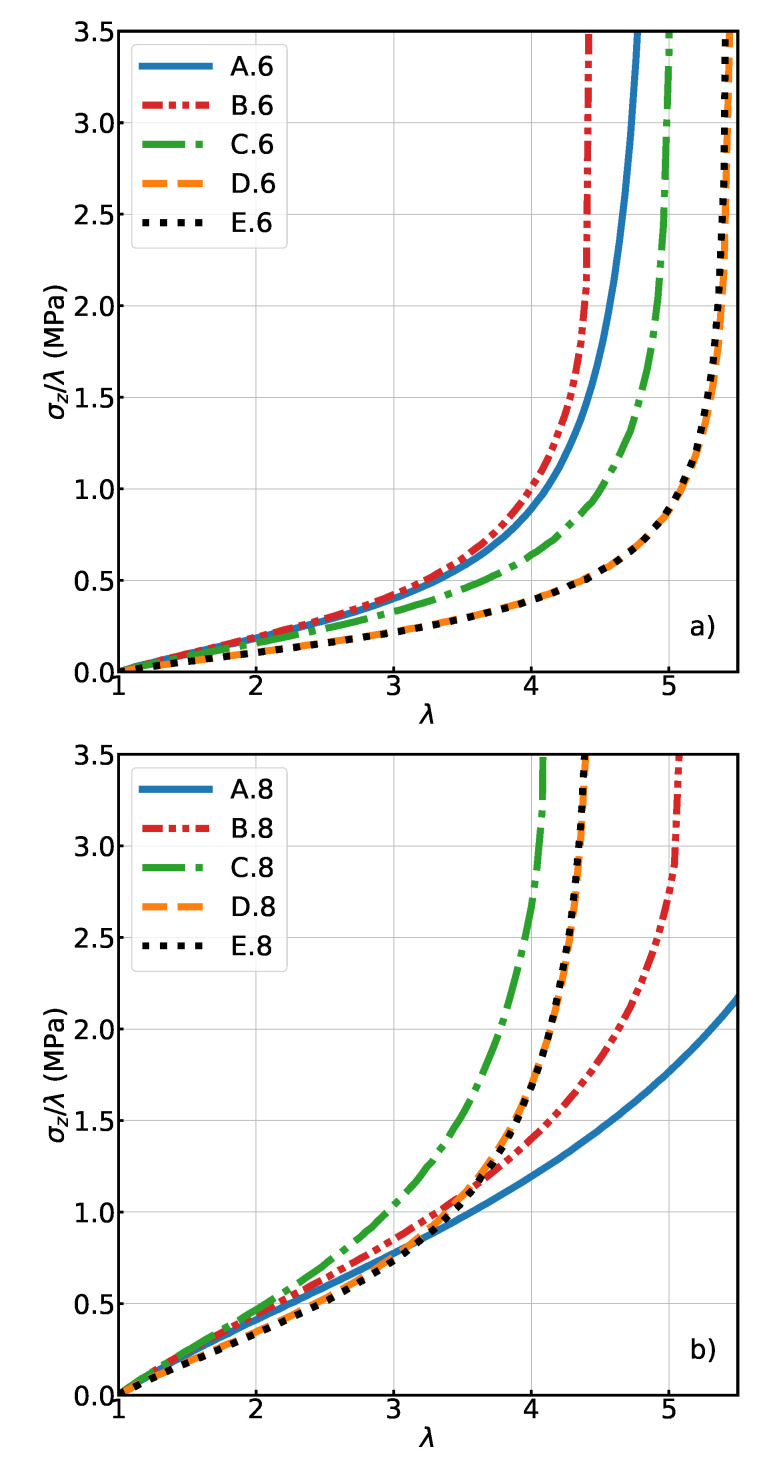
The load vs. stretch diagrams (uni-axial deformation) for systems of (**a**) with connectivity 6 and (**b**) with connectivity 8. (see Table 1 for a complete description of the labels.)

**Figure 4:**
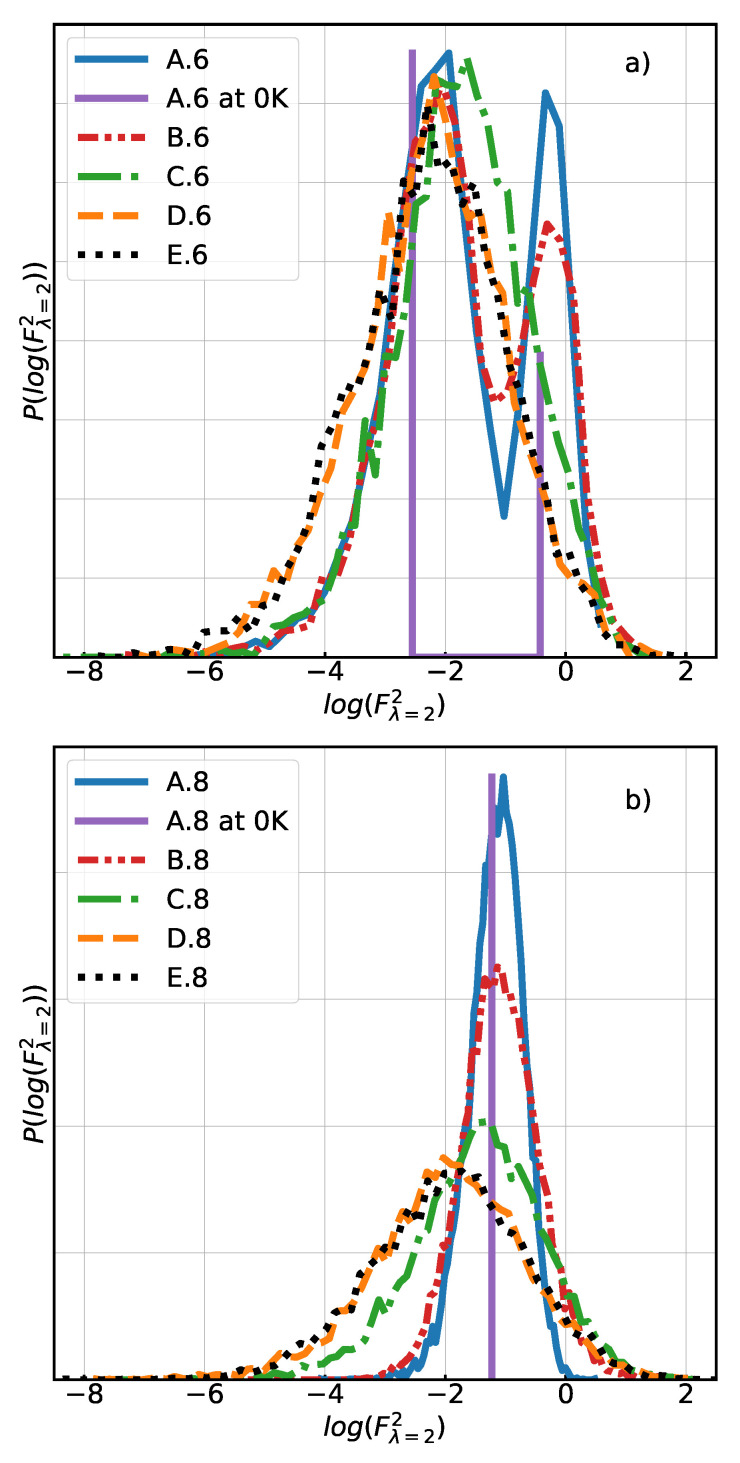
Comparison of distributions of the logarithm of squared forces at strain value equal to 2 (100%) for systems of (**a**) with connectivity 6 and (**b**) with connectivity 8. (Force unit is expressed in J·nm${}^{-1}$. See Table 1 for a complete description of the labels.)

On the other hand, the introduction of polydispersity has a noticeable impact on the finite extension and the stress modulus at high strain values (see Figure 3). This result indicates that the introduction of shorter chains prevails over the introduction of longer chains, because they reach their non-linear, finite-extension regime sooner. Meanwhile, the stress modulus at low strain value is not strongly impacted (Figure 3). The distribution of forces (Figure 4) gives a more detailed description of the differences at a strain value equal to 2. It is presented as the pdf $P(\log\left(F^{2}\right))$, which is more convenient than the pdf of the direct forces P(F), and which can for our purposes sufficiently well be characterised by the mean value and standard deviation.

In the case of the “six chain” model, the distribution of forces found in the mono- and polydisperse system (A.6 and B.6) are quite similar even in the low strain state (Figure 4a). The bimodal shape of the force distribution is due to the alignment of one third of the chains along the stretch axis in the initial state (simple cubic lattice). In A.6, the width of the peaks is only due to the random noise of the nodes, i.e., the thermal fluctuations. In contrast, for the “eight chain” model the distributions of forces show a much clearer difference on adding the polydispersity (A.8 vs. B.8). This distribution remains unimodal, because in the body centered cubic lattice all chains have the same tilt angle with respect to the stretch axis. At low strain, some chains experience a larger force in B.8 than in A.8 (Figure 4b). This is reflected in Table 1 by a small increase in the mean and standard deviation of the force distribution when comparing systems A.8 and B.8, and is also the reason why the B.8 curve has a slightly higher modulus than the A.8 curve in Figure 3 at small deformations.

In making the systems polydisperse, some of the percolating paths will have fewer monomers. These paths are the ones that will get more tight and appear typically in the right tail of the distribution of the forces. Of course this is balanced by some other paths having more monomers and which are therefore less restrictive. However, on average the shorter chains and paths will dominate the stress behaviour, because of the non-linearity of the forces caused by the finite extension. This explains why the modulus increases and the maximum extension decreases. The reason why the impact of polydispersity is more significant in the eight chain system is found in the larger number of short percolating paths in this geometry (and this orientation) compared to the six chain system. We estimate that the introduction of polydispersity leads to a decrease of the length of the shortest percolating path by about 13% in the six chain system and by about 37% in the eight chain system. The details of this estimation are provided in the SI.

These results highlight the relevance of the “mean number of monomers” used in several micro-mechanical models [19,21,22]. Indeed, the addition of a distribution of monomers per chain does not have a strong impact on the stress modulus at low strain for the two models. However, in the literature, the main goal of this kind of analytical model is to produce a good approximation of experimental strain/stress curves valid up to high strain values [22]. With these results, we can easily understand one of the reasons why it is difficult to capture the experimental stress/strain behavior across the full strain range. It is the realisation that there is a “mean number of monomers” that will reproduce experimental data at low strain values, but a lower “mean number of monomers” will be required to describe the experimental behavior at high strain values.

Models with explicit chains of variable lengths, as we have used in this work, give an additional freedom in manipulating the force distributions and can be used to supply input to models, such as the fuse model, to study rupture [29].

### 3.2. Network Topology

The second type of heterogeneity we want to explore is the network topology. To this end we compare two types of random topology networks with our two reference systems. One preserving the structure of the cross-links (simple cubic or body centered cubic) and the other having a random spatial arrangement of the topological constraints.

We first discuss the comparison between the two references (A.6 and A.8) and random networks with a preserved cross-links structure (C.6 and C.8). To generate these systems, we start with the regular six chain and eight chain networks. While keeping a constant number of monomers per chains (monodisperse chains) that is sufficient to allow topological rearrangements beyond first neighbors, we apply the procedure to relax topology. In other words, the node positions are fixed on the regular lattice during the procedure, but the topology of the network changes gradually from a highly symmetric to a random topology. During the relaxation of the topology, heterogeneity is progressively introduced by allowing modifications in the neighbor connections controlled by (3). Figure S4 shows the variation of stress/strain when systems are progressively disordered, from A.6 to C.6 and from A.8 to C.8.

**Figure 5:**
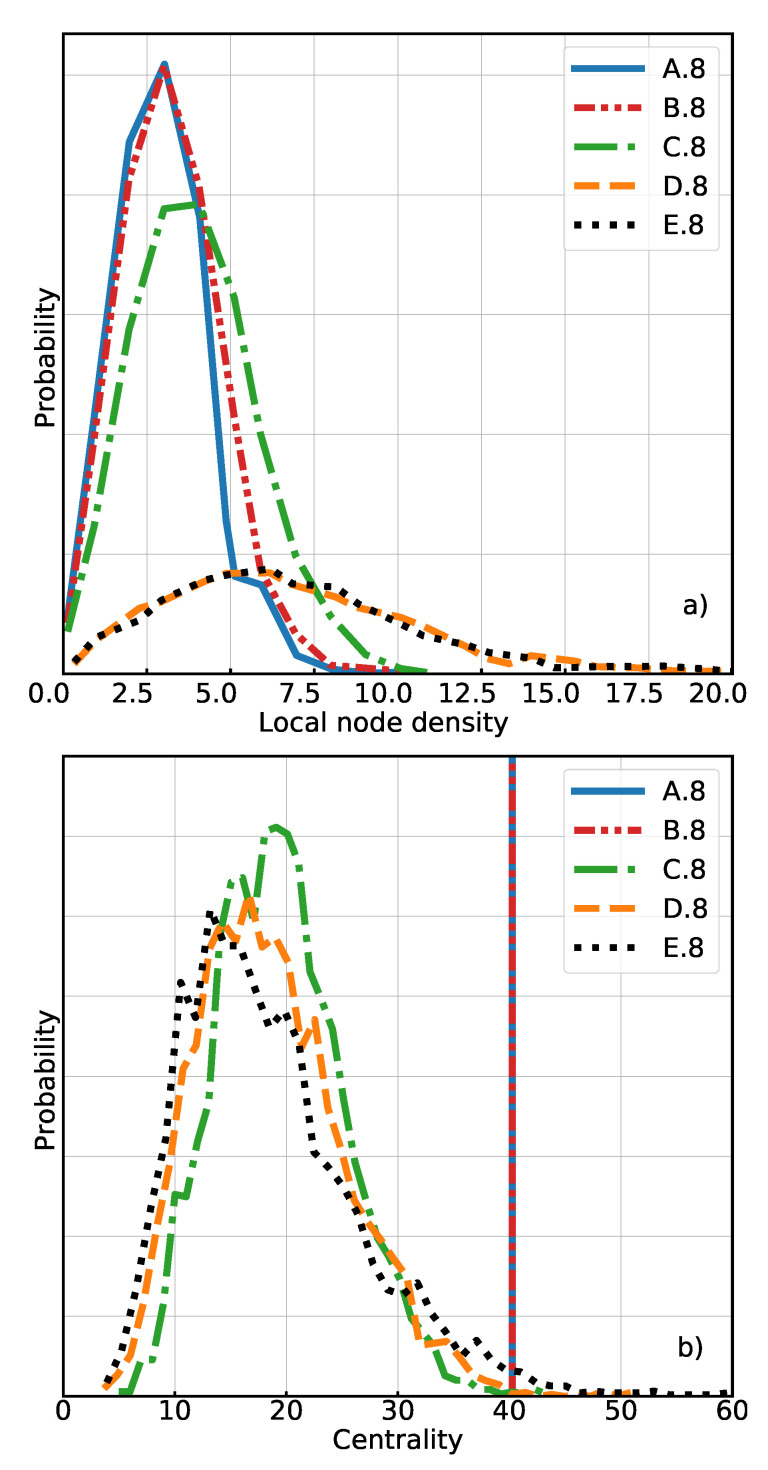
The probability distributions of the (**a**) local node density and (**b**) centrality of networks with a mean node connectivity equal to 8.

The mean centrality value for C.6 and C.8 is smaller than for the references A.6 and A.8. This means that the topology of these systems is clearly different and, given the large standard deviations of centrality in the random networks, also the nodes can no longer be considered to be equivalent. However, the local node density (mean and standard deviation) remains close to that of the reference systems, at least much closer than the fully random networks (D.6 and D.8, see Table 1 and Figure 5a). This suggests that a random topology only has a limited impact on the initial positions of cross-links.

In the case of a “six chain” structure, the introduced topological heterogeneities result in a lower stress modulus compared to the reference, including at low strain values, and in a higher finite extension. Accordingly, the mean value and standard deviation of the distribution of forces are smaller for C.6 system compared to reference A.6 at λ=1 and 2. This can be explained by a reduction in the fraction of chains that are aligned along the stretch axis (Figure S5). These chains are the ones that support most of the stress, because they cannot rotate to adapt to the imposed deformation.

**Figure 6:**
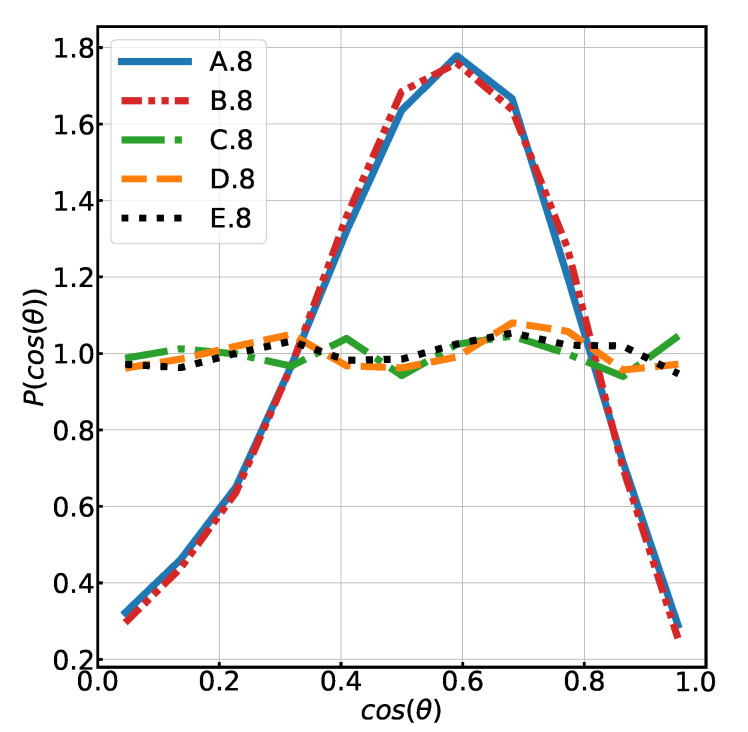
Histogram of the chain orientations at λ=1 (after mechanical relaxation) for systems with a mean node connectivity equal to 8. θ is the tilt angle of the chains with respect to stretch axis. For the systems C.8 to E.8, the orientation of chain orientation is isotropic. The corresponding figure for connectivity 6 is shown in the SI.

In contrast, the introduced topological heterogeneities in the “eight chain” structure system result in a higher stress modulus compared to the reference, even at low strain values, and in a shorter finite extension. Despite a lower mean value of the force distribution in the C.8 system compared to A.8, the higher corresponding standard deviations at λ=1 and 2 show that a non-negligible number of more extended chains exists. Figure 4b shows a significant wing on the right side of the distribution of forces, comparable to that of the B.8 system. This is explained by the larger proportion of chains that are aligned along the stretch axis (see Figure 6) and is in line with the observation that the total stress is mostly due to highly stretched chains forces in this direction.

As a side note, we like to point out that our proposed model allows us to stop the equilibration of topology at any point, which gives an additional control on the degree of the introduced heterogeneity.

### 3.3. Spatial Distribution

Given the observations made above, that the local density of topological constraints is not majorly affected by going to a random topology, we now compare these networks with monodisperse networks where both the positions and the topology are random. This case is illustrated by comparing the D.6 and D.8 systems with, respectively, the C.6 and C.8 models. In particular, we examine whether the behavior of a random network built from structured node positions is equivalent (after dynamic equilibration) with a totally random network.

Figure 3 shows that both systems C (regular initial structure, random topology) are, from a macroscopic point of view, significantly different from the systems D (fully random). The modulus at small deformations is lower in the case of the random initial structure.

Considering the indicators of heterogeneity of systems C.6 and D.6 listed in Table 1, we find that the mean values of centrality are unexpectedly close, and the standard deviation of centrality is just a little larger in the full random network D.6. From this indicator point of view, these two networks are very similar. The chain orientation is also almost isotropic after mechanical relaxation of both systems D.6 and C.6. On the other hand, the local node density is quite different and is significantly higher when the initial structure is fully random. The same is true for the standard deviation of the local density. This means, that in the D.6 system, nodes are on average closer to each other in the unstretched state than in C.6 (or than in the A.6 reference), and that the density is more heterogeneous, as expected. As a consequence of this spatial node distribution, the mean of the distribution of forces in the relaxed state is lower in D.6 than in C.6, while the standard deviation is almost the same. This behaviour remains valid also for higher strains.

Similar observations can be made for the higher connectivity “eight chain” systems (see Table 1). Again, the distribution of centrality is similar for C.8 (regular initial structure) and D.8 (fully random), as can be seen in Figure 5b. Moreover, there is no substantial difference between system C.8 and D.8 in terms of chain orientation (Figure 6), both of them are isotropic at rest. The standard deviation and mean of the centrality are only slightly higher in the fully random system. If we compare the local node densities, however, we again find the same big difference (Figure 5a) between C.8 and D.8, the nodes being more packed on average and more heterogeneously distributed in the latter system. The distribution of forces shows the same tendency as in the “six chain” case, in the relaxed state as well as under strain.

Like in the previous subsection, it is also here possible to change progressively from the fully regular initial structure (systems C) to the fully random initial structure (systems D). This is achieved by assigning the nodes initially to regular lattice sites and adding a random displacement with increasing amplitude before connecting the network. Figure S6 shows the variation of stress/strain when systems are progressively disordered, from C.6 to D.6 and from C.8 to D.8.

The fact that the random networks are less stiff than the more regular networks, could be explained by the notion that the regions with higher density of nodes in the initial structure tend to form clusters of more connected nodes, while at the same time the clusters are connected to each others by fewer chains. The initial local density therefore affects the node centrality. This intuitive idea is confirmed by the observation that the local node density after mechanical relaxation and the centrality are partly correlated (see SI).

**Figure 7:**
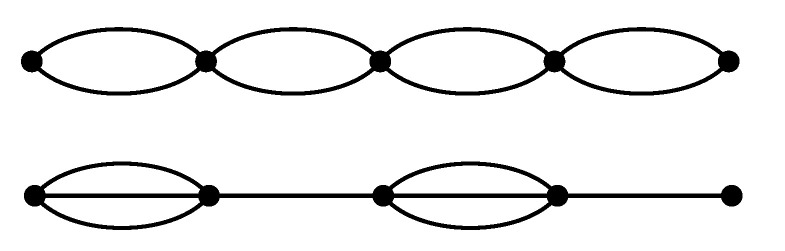
Toy model of a chain of springs in 1-D. Each line represents a spring of equal stiffness *k*. The more heterogeneous bottom system with stiffer clusters is globally less stiff than the regular chain. Note that the connectivity and the number of springs is the same in both cases.

Compared to the systems with regular initial structure, the local stiffness is higher in clusters and is lower in the surrounding areas. On average, however, the global stiffness decreases. This argument can be illustrated by considering a 1-D toy model sketched in Figure 7 of two different structures. If each spring has the same stiffness *k*, then the regular 2-2 chain has a global stiffness *k*, while the “cluster” chain 3-1 has a global stiffness 3k/4<k. This effect could be even enhanced in 3-D due to possible chain reorientations.

### 3.4. Connectivity

There are various studies on defects in polymer networks [14,49] or the effect of cross-link connectivity [8]. It is also a well-known fact that during the chemical reticulation procedure the maximal connectivity of the cross-linker agent is not always reached, which results in a lower average value of connectivity. In this context, we now compare ideal systems (same connectivity for each node) with systems for which the functionality of the cross-links are not uniform. We introduce this type of heterogeneity by selecting some nodes and increasing or decreasing their connectivity, in such a way that the distribution of node connectivity becomes triangular ranging from 4 to 8 with a mean value of 6 connections (system E.6), or the range 6 to 10 with mean of 8 (system E.8). These nodes are randomly connected by monodisperse chains using the aforementioned procedure.

The mechanical response of these systems is shown in Figure 3. Interestingly, the effect of a distributed connectivity appears to be negligible in both cases. In other words, the mechanical response only depends, with respect to this property, on the mean connectivity number. The dispersion around this number does not significantly change the mechanical behavior.

### 3.5. Connections beyond First Neighbors

**Figure 8:**
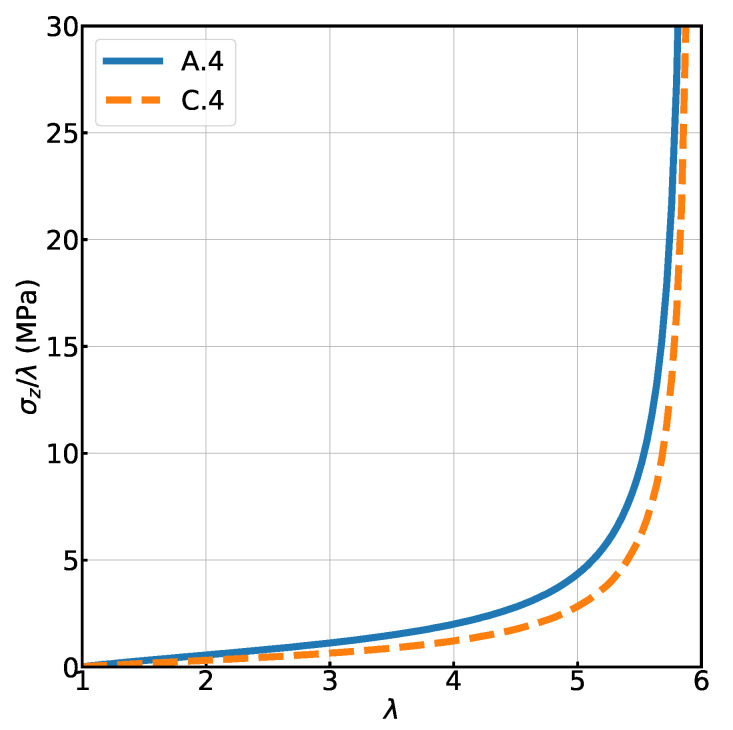
Comparison of the load versus stretch diagrams (uni axial deformation) of the two different 4 chain networks.

The last issue we like to address is whether the differences between systems A (regular lattice) and C (random topology with a regular structure) are due to the possibility for chains to connect to nodes beyond first neighbors, thus changing the initial end-to-end distance. In order to investigate this, we prepared a system A.4 on a regular body centered lattice, where each node has eight nearest neighbors, but is only connected to 4 of them, forming a double diamond lattice. The system C.4 has the same initial positions, but the topology was randomized using the procedure that was introduced before, while forcing the nodes to connect to nearest neighbors only. By doing so, we ensure that the end-to-end distance for every chain is identical at the end of generation procedure for both networks. As shown in Figure 8, the random topology affects the uni-axial mechanical response of networks and is the cause of a lower modulus. Contrary to the A and B systems, the finite extension $\lambda_{max}$ is the same for the random topology C.4 and its reference model A.4. This could, however, also be due to the fact that the orientation of the chains relative to the elongation axis is different in the systems with connectivity 6 and 8.

At zero-strain the mean values and standard deviations of the distributions of forces are already different for A.4 and C.4 (see Table 1). The difference is comparable to the one observed in systems with connectivity 6 and 8, in that despite a higher standard deviation of forces in C.4, the average value is small enough to have a large fraction of chains with smaller force than in the A.4 reference system. This tendency persists at low and high strain values, resulting in the stress–strain curve shown in Figure 8. Like in the other systems where we have added heterogeneity, the mean centrality decreases from A.4 to C.4, but in this case the standard deviation is large enough to include the mean centrality of the reference system.

The most interesting quantity, however, is the local node density. The heterogeneous topology that was introduced during the generation of the network C.4, has an effect on the spatial distribution of nodes after the mechanical equilibration step. As in the systems with connectivity 6 and 8, the C.4 system with a random topology has a higher mean and standard deviation of the local node density than the A.4 reference system, which means that the nodes are closer to each other and more heterogeneously distributed. However, the difference is significantly larger. This is somewhat surprising, because C.4 and A.4, with equal initial distances, should be more similar than C.6 and A.6 or C.8 and A.8. The larger effect in systems of connectivity 4 is possibly due to the lower connectivity of these systems, which enhances the sensitivity to local perturbations of the network.

It therefore seems that all of the effects that were observed in changing from A.6 to C.6 or from A.8 to C.8 are still present without connections beyond the first neighbors, except perhaps for the variation in finite extension.

## 4. Discussion

There are a number of different causes that can result in disorder and heterogeneities in polymer networks. In general, all of them have an impact on the average macroscopic mechanical behavior of the network, i.e., the stress–strain curve with respect to modulus and finite extension. The main conclusions that we can draw from this study are the following:

- Regular lattices can have particular properties due to their anisotropy [50]. Cubic structures are anisotropic, because they are sensitive to the direction of strain with respect to the lattice vectors.
- On introducing more heterogeneities in the network, the force distribution becomes wider. However, heterogeneities do not have a systematic effect on the finite extension and modulus. Indeed, depending on the initial structure, introducing heterogeneity can impact the mechanical properties in one way or the other.
- More surprisingly, networks with a similar topology and isotropy can have a significantly different mechanical response (C.6 and C.8 vs. D.6 and D.8). In particular, it is possible to decrease the small-strain modulus while keeping the same finite extension (C.8 vs. D.8). The difference seems to originate from the spatial arrangement of the reticulation nodes (local node density), as indicated by the similarity between the systems C and D in terms of the centrality and the chain orientation at zero-strain. The system is sensitive to, and has a memory of, the position of the nodes and their “well-dispersedness” at the time that the reticulations have been created. The way the networks are generated and the descriptors by which they are characterized are therefore essential. This might serve as an experimental lever to adjust the mechanical response of a polymer network.
- Finally and unexpectedly, the variability of the cross-link valency has no impact at all, neither on the macroscopic mechanical behavior, nor on the local force heterogeneity, provided that the average valency is kept fixed.

We have demonstrated that to some extent it is possible to generate networks with controlled activation of different sources of heterogeneity. The model was kept simple on purpose and lacks some essential features of realistic polymer networks, in particular entanglements and chain breaking. However, we believe that this kind of approach can enhance our understanding by separating the contributions of each source of heterogeneity to the global disorder and the average mechanical response that are observed in real systems, where all those effects are combined.

The simulated polymer networks such as we have described here can be made more realistic in order to feed higher-scale simulation methods, like finite elements or fuse networks. For such realistic models, the Kuhn length, mass density of monomers, number density and valency of the cross-links, entanglement mass and chain breaking energy can generally be obtained experimentally. However, as we have shown, this is not sufficient to build a network that is capable of simulating a realistic mechanical response. Indeed, it is also important to know the structure of the network and to generate it realistically.

## 5. Conclusions

We have demonstrated the relevance of a simple numerical model of polymer networks to investigate how much different sources of heterogeneity impact the mechanical response at the microscopic and at the microscopic scale.

Starting from ideal regular lattices, heterogeneities have been progressively introduced until fully random networks were obtained. As expected, the distribution of forces gets broader when more and more heterogeneity is introduced. We found that the heterogeneity of local stress is mostly impacted by heterogeneity in the topology (graph notion) while the average macroscopic response is mostly impacted by heterogeneity in the initial structure (spatial notion). Surprisingly, the non-uniformity of crosslink connectivity has no significant impact (only the average connectivity matters).

These results can provide insight for experimentalists who wish to foresee how much changing the process of production of the polymer networks will impact (or not) the distribution of their mechanical properties.

For numerical simulations, our results also highlight the importance of the way the network is generated and characterized. In particular, networks with similar topology but generated from different structures can have significantly different mechanical responses.

## Figures and Tables

**Table 1:** Summary of the simulated system and results. The characteristics of the networks, as well as the different indicators to characterize them, are given. The average values are listed including the non-zero standard deviations.

System	A.6	B.6	C.6	D.6	E.6	A.4
topology	six chain	six chain	random	random	random	4 chains
structure	six chain	six chain	six chain	random	random	eight chain
chainlength	27	27±5	27	27	27	12
connectivity	6	6	6	6	6±2	4
centrality	11.85	11.85	8.31±1.99	8.29±2.74	8.32±3.35	4.60
localnodedensity	3.12±1.44	3.14±1.49	4.13±1.98	7.73±4.19	7.72±4.05	3.45±1.53
$\log(F_{\lambda =1}^{2})$	−1.8±0.6	−1.8±0.7	−2.0±1.2	−2.5±1.2	−2.6±1.2	−0.9±0.8
$\log(F_{\lambda =2}^{2})$	−1.7±1.2	−1.7±1.2	−1.8±1.1	−2.3±1.3	−2.3±1.3	−0.3±0.7
System	**A.8**	**B.8**	**C.8**	**D.8**	**E.8**	**C.4**
topology	eight chain	eight chain	random	random	random	random
structure	eight chain	eight chain	eight chain	random	random	eight chain
chainlength	22	22±5	22	22	22	12
connectivity	8	8	8	8	8±2	4
centrality	40.26	40.26	19.48±5.72	18.98±7.27	18.69±8.22	4.00±0.71
localnodedensity	3.05±1.46	3.18±1.51	3.92±1.86	6.91±3.85	6.91±3.96	5.38±2.90
$\log(F_{\lambda =1}^{2})$	−1.7±0.6	−1.6±0.7	−1.8±0.9	−2.3±1.1	−2.3±1.1	−1.5±1.1
$\log(F_{\lambda =2}^{2})$	−1.2±0.5	−1.1±0.7	−1.5±1.1	−2.0±1.3	−1.9±1.3	−1.2±1.2

## Data Availability

The data presented in this study are available on request from the corresponding author.

## Supplementary Materials

The following are available online at https://www.mdpi.com/2073-4360/13/5/757/s1.
