# Supplementary material for: Heterogeneity Effects in Highly Cross-Linked Polymer Networks
Source: Polymers (Basel). 2021 Feb 28;13(5):757. doi: 10.3390/polym13050757 (PMC7957597; doi:10.3390/polym13050757)
Supplement: Supplementary file 1 [file polymers-13-00757-s001.pdf]

Supplementary Materials: Heterogeneity Effects in Highly Cross-linked Polymer Networks

Gérald Munoz [1], Alain Dequidt [2,*], Nicolas Martzel [1,*], Ronald Blaak [2], Florent Goujon [2], Julien Devémy [2], Sébastien Garruchet [1], Benoit Latour [1], Etienne Munch [1], Patrice Malfreyt [2]

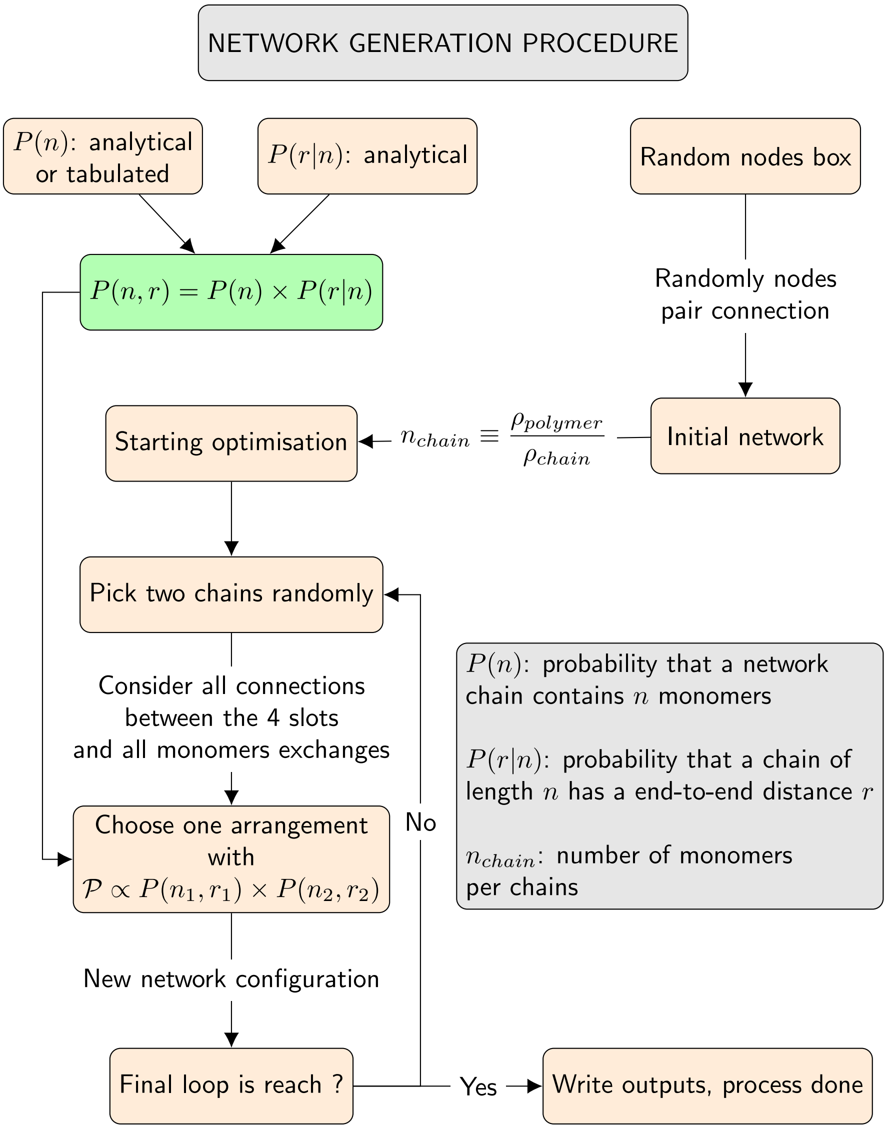

Figure S1. Algorithm for the equilibration of topology.

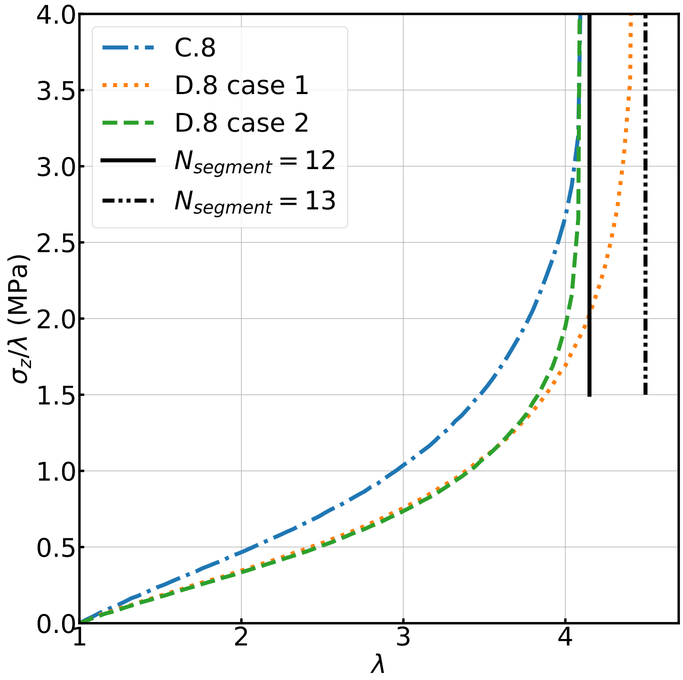

Figure S2. Examples of maximal strain due to the length of shortest percolating path. The two vertical lines represent maximal theoretical strain value when the shortest path consists of 12 or 13 segments.

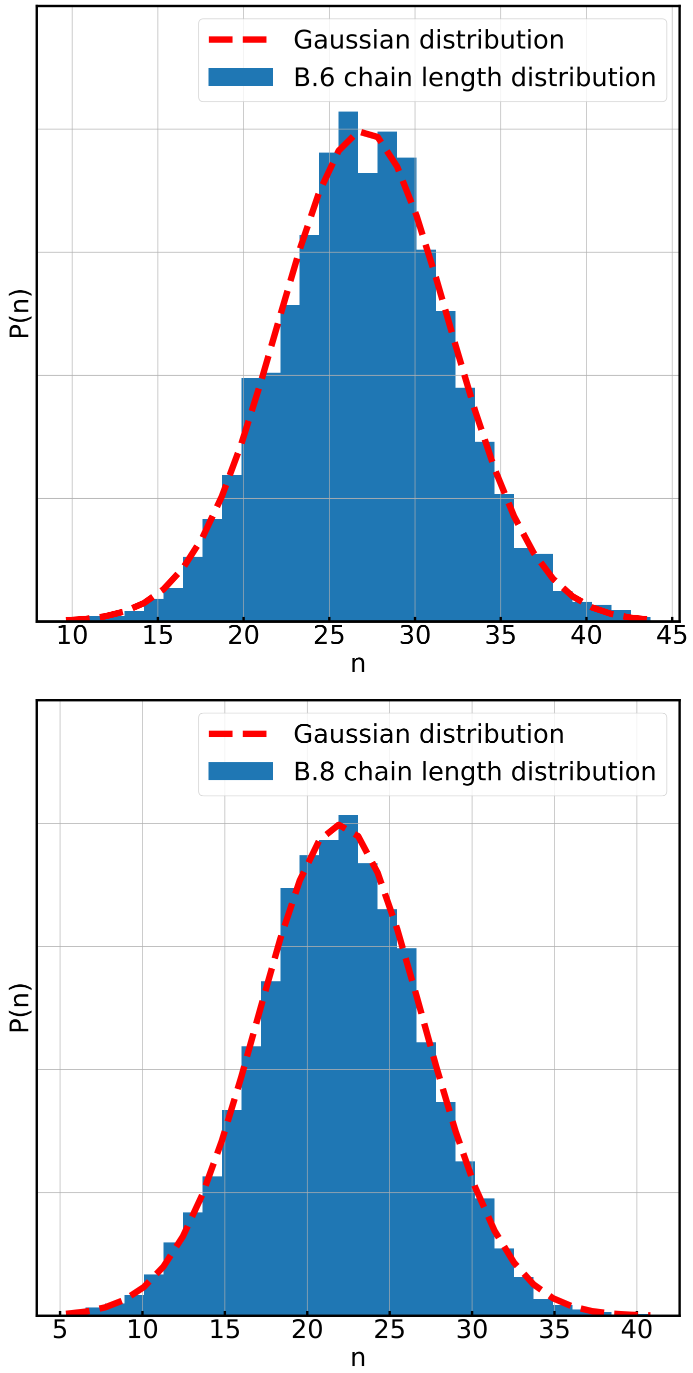

Figure S3. Verification of the consistency between target shape of the distribution of number of monomers per chain $P(n)$ (dashed red) and simulation sample after equilibration of the topology (blue histogram). Top: $n = 27 \pm 5$, bottom: $n = 22 \pm 5$.

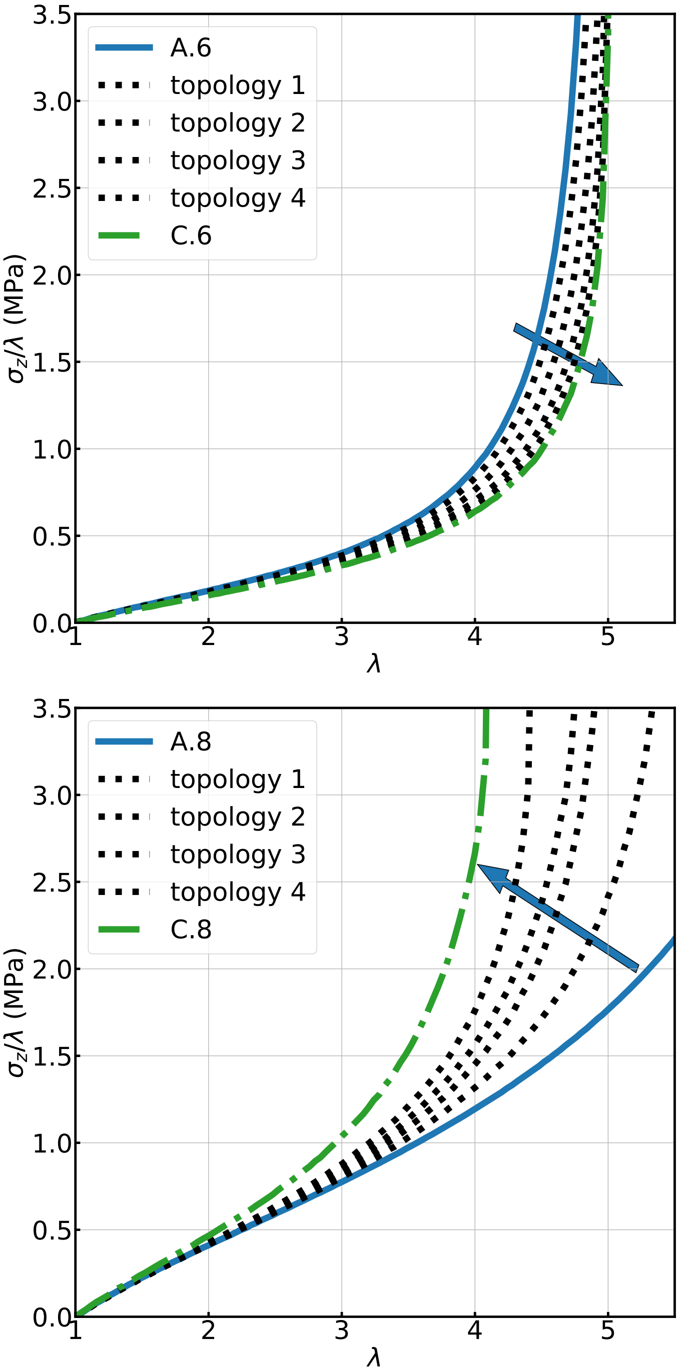

Figure S4. Examples of stress/strain curve for intermediate topologies between systems A and C. The arrow indicates the direction of an increasing number of steps during the topology equilibration process.

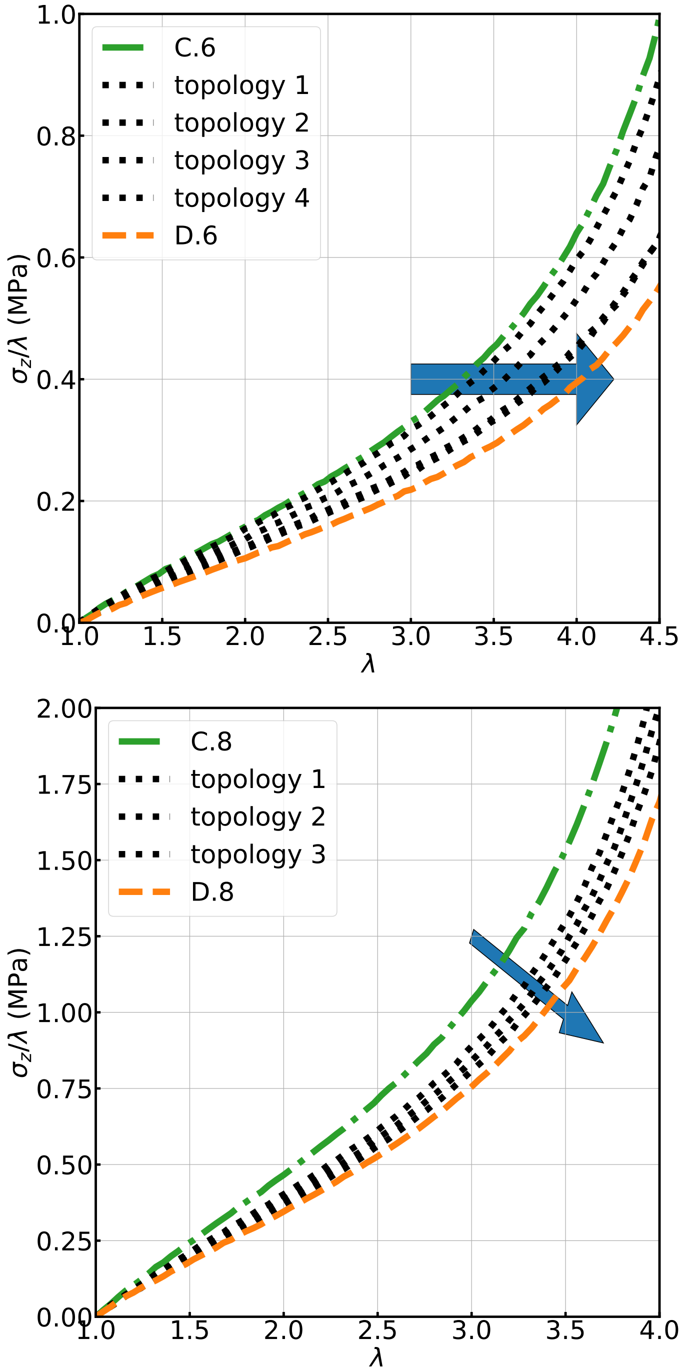

Figure S5. Examples of stress/strain curve for intermediate topologies between systems C and D. The arrow indicates the direction of an increasing number of steps during the topology equilibration process.

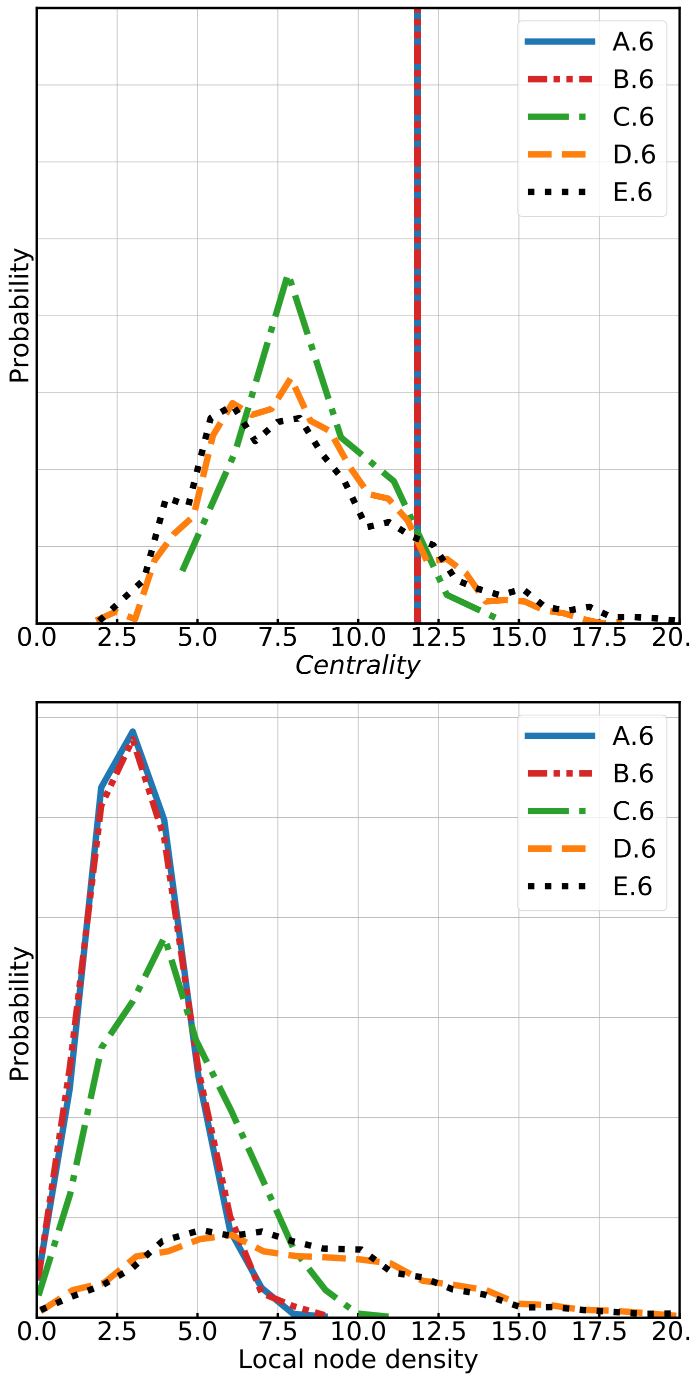

Figure S6. Local node density and centrality of networks with mean node connectivity equal to 6

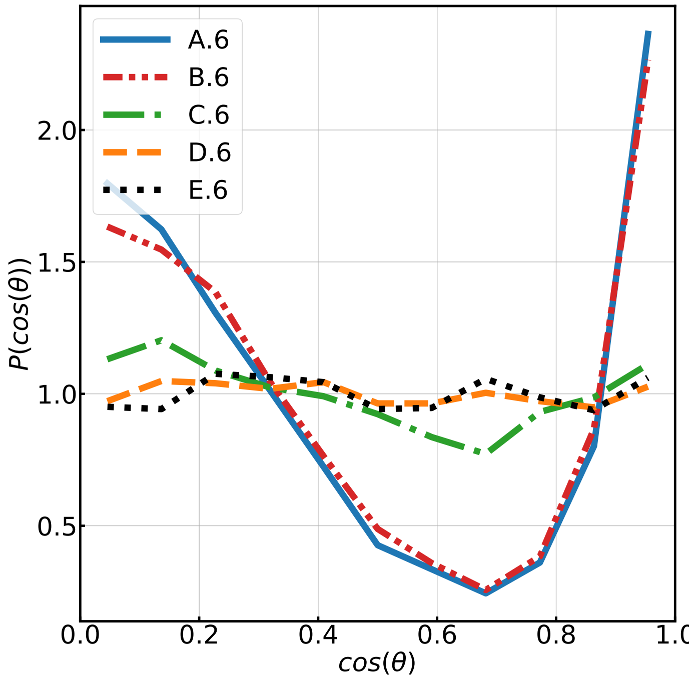

Figure S7. Histogram of the chain orientations at $\lambda = 1$ (after mechanical relaxation) for systems with mean node connectivity equal to 6. θ is the tilt angle of the chains with respect to axis x. For systems C.6 to E.6, the distribution of chain orientation is almost isotropic.

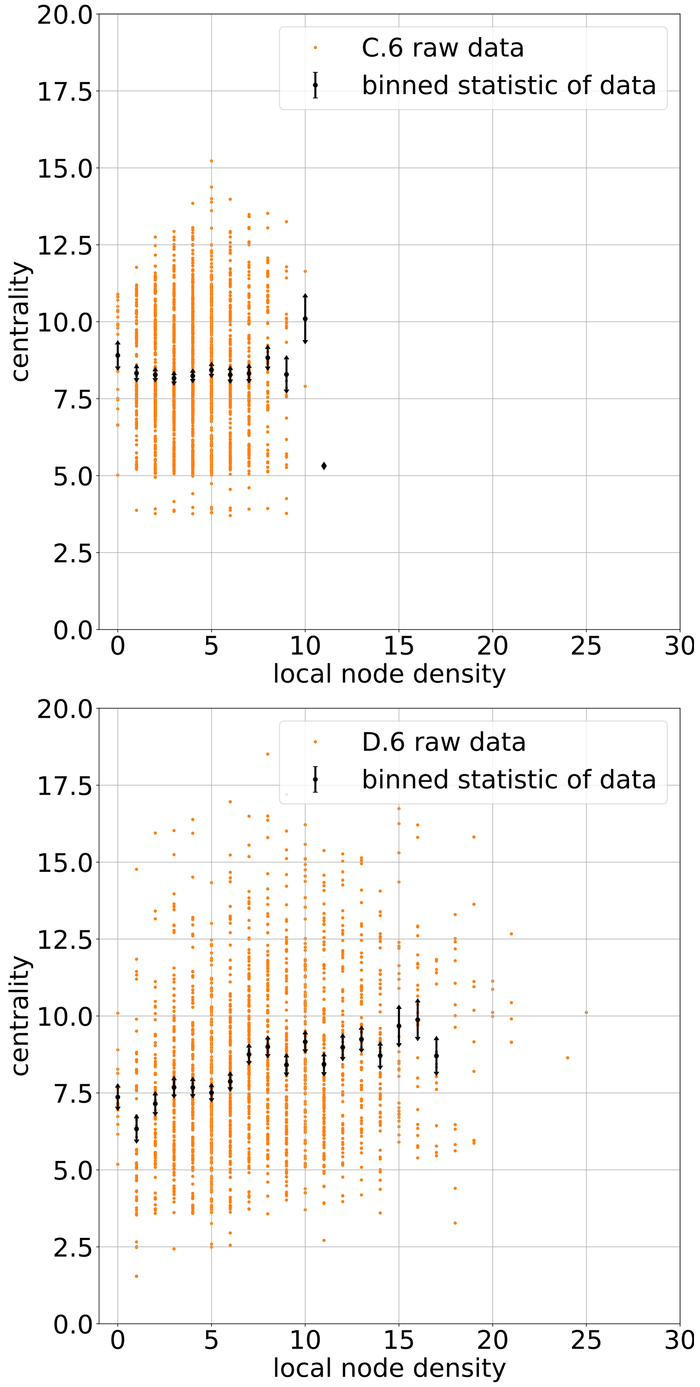

Figure S8. Correlations between the centrality and the local node density for systems with mean connectivity 6. The error bar is the standard error of the mean. For C.6 (regular structure, random topology), no clear correlation. For D.6 (random structure and topology) the centrality of the nodes is on average positively correlated with the local node density.

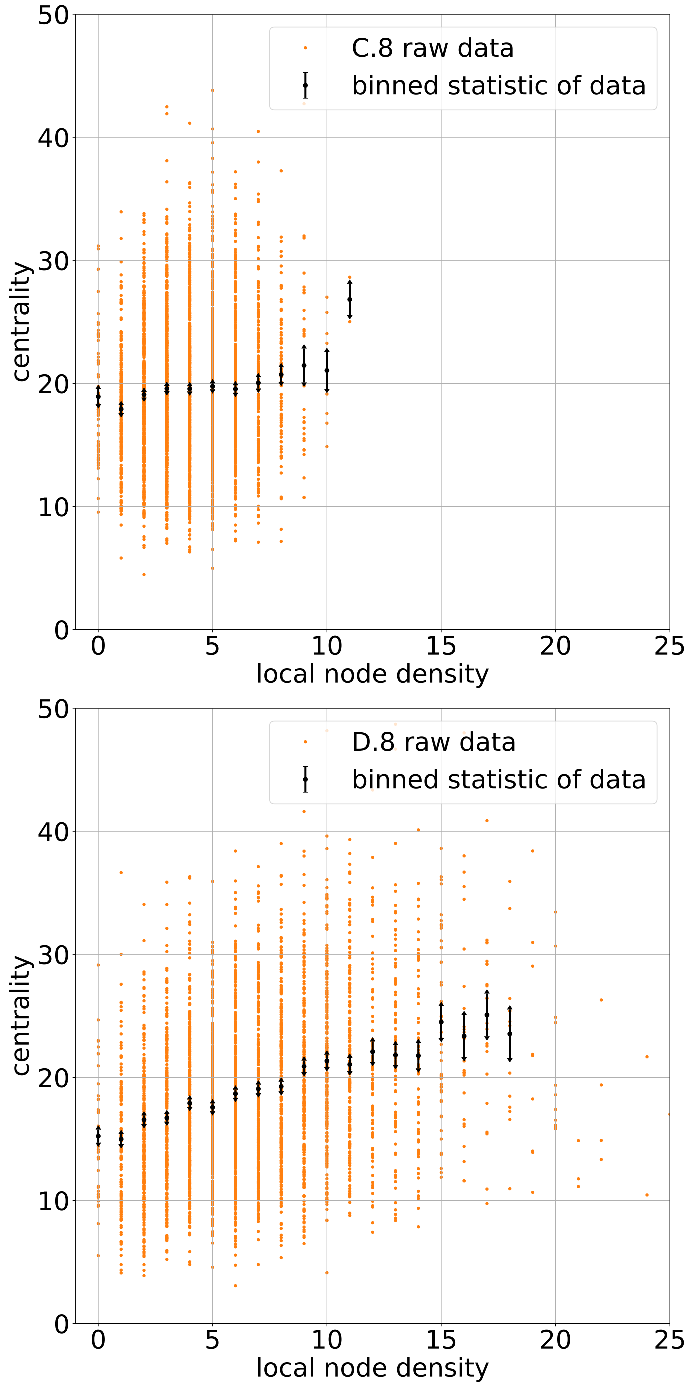

Figure S9. Correlations between the centrality and the local node density for systems with mean connectivity 8. The error bar is the standard error of the mean. The same remarks hold as for connectivity 6. For C.8 (regular structure, random topology), no clear correlation. For D.8 (random structure and topology) the centrality of the nodes is on average positively correlated with the local node density.

1. Estimation of the maximal extension for regular lattices of polydisperse chains

We make the following approximations or assumptions for estimating the maximal extension in our periodic boxes:

- The maximal extension is the length of the shortest path (in number of monomers) connecting any node to its first image in the direction of extension. This means that we do not consider that the possibility of direct self-connection beyond the first periodic image could be more restrictive.
- The shortest path is one of the percolating paths having the smallest number of chains. This means that we do not consider the possibility that the shortest path would have more chains but fewer monomers in total.
- The probability $p(l)$ for a path to have a length l is independent of the probability of length of the other paths. l will also be considered as a continuous variable.

Let us call n the smallest number of chains required to percolate across the box in the direction of extension, and N the number of such percolating paths having n chains. l_0 will be the average length of the chains and σ their standard deviation.

The total length of a path of n chains is a sum of n normal variates. Its distribution is[1]

$$p(l) = \left(2\,n\,\pi\,\sigma^2\right)^{-1/2} \exp\left(-\frac{1}{2\,n}\left(\frac{l - n\,l_0}{\sigma}\right)^2\right) \tag{1}$$

The probability for a path to have a length no shorter than L is

$$p(l \not< L) = \int_L^\infty p(l')\,\mathrm{d}l' = \frac{1}{2}\,\mathrm{erfc}\left(\frac{L - n\,l_0}{\sqrt{2\,n}\,\sigma}\right) \tag{2}$$

with erfc the complementary error function. The probability that, among the N shortest path candidates, none is shorter than L is (assuming independent probabilities)

$$p(\min l > L) = \left(\frac{1}{2}\,\mathrm{erfc}\left(\frac{L - n\,l_0}{\sqrt{2\,n}\,\sigma}\right)\right)^N \tag{3}$$

So finally, the probability that the shortest path has length L is

$$p(l_{\min} = L) = -\frac{\mathrm{d}}{\mathrm{d}L}\left(\left(\frac{1}{2}\,\mathrm{erfc}\left(\frac{L - n\,l_0}{\sqrt{2\,n}\,\sigma}\right)\right)^N\right) \tag{4}$$

The most probable value of $l_{\min}$ is such that $\frac{\mathrm{d}p(l_{\min}=L)}{\mathrm{d}L} = 0$. So we have to solve

$$\frac{\mathrm{d}^2}{\mathrm{d}L^2}\left(\left(\frac{1}{2}\,\mathrm{erfc}\left(\frac{L - n\,l_0}{\sqrt{2\,n}\,\sigma}\right)\right)^N\right) = 0 \tag{5}$$

This equation is solved numerically.

The size and geometry of System B.6 is such that $n = 12$ and $N = 144$, $l_0 = 27$ (Kuhn units) and $\sigma = 5$. We find $l_{\min} \approx 281$, which is about 13 % shorter than in System A.6 ($l_{\min} = 324$).

In System B.8, $n = 24$, $l_0 = 22$ and $\sigma = 5$. But N is much higher [2]. Indeed,

$$N = 144 \times \frac{(n\,!)^2}{((n/2)\,!)^4} \approx 10^{12}\,. \tag{6}$$

In this case, $l_{\min} \approx 333$, which is about 37 % smaller than in System A.8 ($l_{\min} = 528$).

[1] This would also be true if the chain lengths are not normally distributed provided $n \gg 1$, in accordance with the central limit theorem.

[2] In the 111 direction, the opposite would have been true. N would have been much greater in B.6 than in B.8.
